# Supplementary material for: Creating a culture, not just a space—A qualitative investigation into reflective practice groups in inpatient mental health settings from the perspectives of facilitators and attendees
Source: PLoS One. 2025 Jan 3;20(1):e0316030. doi: 10.1371/journal.pone.0316030 (PMC11698324; doi:10.1371/journal.pone.0316030)
Supplement: S1 File — (DOCX) [file pone.0316030.s001.docx]

**Interview Guides**

**Interview Schedule – Clinical Psychologists**

**Briefing:**

- Confirm that the consent form has been signed.
- Thank participants for agreeing to take part in the interview.
- Explain the aim of interview – to find out more about their experiences on reflective groups.
- Reassure the participant that they could openly talk about their experiences, there is no right or wrong, preferred or not preferred answer.
- Remind that the participant could feel free to take a break at any time during the interview.

**Introduction:**

1. Can you tell me about your experiences of facilitating reflective practice session in an inpatient setting?
   - Prompt: what led you to become a reflective practice group facilitator?

**Impact of Reflective Practice Sessions:**

1. From your experiences, what impacts, if any, can reflective practice sessions bring about?
   - Prompt: how has facilitating reflective practice sessions impacted you personally and professionally?
   - Prompt: what impact has it had on your team?
   - Prompt: what impact has it had on your team members individually?
   - Prompt: what impact has it had on your relationship with the team?
   - Prompt: what impact has it had on your organization?
2. What are some other things, if any, that you hope reflective practice sessions would bring about?
   - Prompt: personally, professionally, individual team member, at a team-level, and organizational level?

**Organizing and Facilitating in Reflective Practice Sessions:**

1. What are your experiences in organizing a reflective practice session?
   - Prompt: how does it feel? It is easy or challenging?
   - Prompt: other practical challenges?
   - Prompt: preparation, recruitment, and debriefing / reflecting on the reflective practice session?
2. What may be helpful or unhelpful when attempting to organize a reflective practice session?
3. What are your experiences of engaging people in a reflective practice session?
   - Prompt: how does it feel? It is easy or challenging?
4. What may be helpful or unhelpful to engage people in a reflective practice session?
5. What are some other pressures, if any, in organizing or facilitating in reflective practice sessions? How did you manage it?
6. What might be some of the unique challenges or impacts of reflective practice sessions in inpatient settings?
7. Some people run groups for the team in which they work, whilst others have a more specific and ‘outsider’ role. What are your experiences and views on this?
   - Prompt: what are some pros and cons of facilitating reflective practice sessions with your own team?

**Potential Improvements:**

1. Are there things, if any, that could be done to improve the reflective practice sessions?
   - Prompts: before, during, and after the sessions?
   - Prompts: personal, professionally, individual team member, team-level, organizational?

**Ending:**

1. Are there any other aspects of reflective practice sessions that you think are important and we have not discussed?

**Interview Schedule – Inpatient Staff**

**Briefing:**

- Confirm that the consent form has been signed.
- Thank participants for agreeing to take part in the interview.
- Explain the aim of interview – to find out more about their experiences on reflective groups.
- Reassure the participant that they could openly talk about their experiences, there is no right or wrong, preferred or not preferred answer.
- Remind that the participant could feel free to take a break at any time during the interview.

**Introduction:**

1. Can you tell me about your experiences of reflective practice sessions in an inpatient setting?

**Impact of Reflective Practice Sessions:**

1. From your experiences, what impacts, if any, can reflective practice sessions bring about?
   - Prompt: have reflective practice sessions impacted you personally?
   - Prompt: have reflective practice sessions impacted your professional work?

Prompt: have reflective practice sessions impacted the team?

- - Prompt: have reflective practice sessions impacted the organization?

1. What are some other things, if any, that you hope reflective practice sessions would bring about?
   - Prompt: personally, professionally, at a team-level, and at an organizational level?

**Accessing and Engaging in Reflective Practice Sessions:**

1. What are your experiences in trying to attend a reflective practice session?
   - Prompt: what could be some practical barriers?
   - Prompt: how does it feel? Is it easy or challenging?
2. What may make it easier, or harder, for you to attend a reflective practice session?
3. What may make it easier, or harder, for you to engage more in a reflective practice session?
4. What are some other pressures, if any, in attending or engaging in reflective practice sessions? How did you manage it?
5. Some groups are run by a facilitator from the team in which they work together, whilst other groups have an ‘outsider’ facilitator. What are your experiences and views on this?
   - Prompt: what are some pros and cons of having a facilitator from your own team?

**Potential Improvements:**

1. Are there things, if any, that could be done to improve the reflective practice sessions?
   - Prompts: before, during, and after the sessions?
   - Prompts: personal, team-level, organizational?

**Ending:**

1. Are there any other aspects of reflective practice sessions that you think are important and we have not discussed?
